# Fine Tuning the Intermolecular Interactions of Water Clusters Using the Dispersion-Corrected Density Functional Theory

**DOI:** 10.3390/molecules28093834

**Published:** 2023-04-30

**Authors:** Alfonso Ferretti, Laura Canal, Robert A. Sorodoc, Sourab Sinha, Giuseppe Brancato

**Affiliations:** 1 Scuola Normale Superiore and CSGI, Classe di Scienze, Piazza dei Cavalieri 7, I-56126 Pisa, Italy; 2 Istituto Nazionale di Fisica Nucleare (INFN) Sezione di Pisa, Largo Bruno Pontecorvo 3, I-56127 Pisa, Italy; 3 Dipartimento di Ingegneria Civile ed Industriale, Università di Pisa, Largo Lucio Lazzarino 2, I-56124 Pisa, Italy

**Keywords:** density functional theory, water clusters, water intermolecular energy, dispersion corrections, DFT-D4

## Abstract

Dispersion-inclusive density functional theory (DFT) methods have unequivocally demonstrated improved performances with respect to standard DFT approximations for modeling large and extended molecular systems at the quantum mechanical level. Yet, in some cases, disagreements with highly accurate reference calculations, such as CCSD(T) and quantum Monte Carlo (MC) calculations, still remain. Furthermore, the application of general-purpose corrections, such as the popular Grimme’s semi-classical models (DFT-D), to different Kohn–Sham exchange–correlation functionals sometimes leads to variable and inconsistent results, which recommend a careful prior evaluation. In a recent study, we proposed a simple optimization protocol for enhancing the accuracy of these DFT-D methods by following an alternative and system-specific approach. Here, adopting the same computational strategy, we show how the accurate MC intermolecular interactions of a large set of water clusters of variable sizes (i.e., 300 (H_2_O)*_n_* structures, *n* = 9, 15, 27) can be reproduced remarkably well by dispersion-corrected DFT models (i.e., B3LYP-D4, PBE-D4, revPBE(0)-D4) upon re-optimization, reaching a mean absolute error per monomer of ~0.1 kcal/mol. Hence, the obtained results support the use of this procedure for fine-tuning tailored DFT-D models for the accurate description of targeted molecular systems.

## 1. Introduction

The explicit inclusion of dispersion corrections in density functional theory (DFT) approximations has led to a general improvement in the description of aqueous systems of all kinds, ranging from small water clusters to liquid and ice structures [1]. In this regard, different efficient approaches have been proposed, which account for the poor description of the van der Waals interactions within the DFT framework (see, e.g., ref. [2] for a recent review). Among others, the family of DFT-D models proposed by Grimme and coworkers [3,4,5] have emerged as one of the most accurate and computationally efficient approaches for modeling and simulating large molecular systems. The latest updated implementations, termed DFT-D3 [5] and DFT-D4 [6,7], have been widely and successfully tested for calculations on various molecular systems, considering both structural and thermochemical properties [8,9]. Moreover, trustworthy DFT-D*x* models can be conveniently employed for building up datasets to be used for training and testing advanced machine learning potentials [10]. Yet, different dispersion-inclusive DFT models may lead to results that are not necessarily consistent with one another, making the choice of the best performing method not always obvious. For instance, recent comparative studies on water systems using dispersion-corrected and uncorrected DFT methods have evidenced a highly variable agreement with respect to reference data [1,11]. The non-trivial analysis of the observed deviations from the accurate benchmark results has identified several possible causes for this, among which are inconsistencies in the monomer polarizabilities and deformation energies, a poor description of the dispersion and exchange-overlap interactions, and additional many-body errors [1]. However, the main contribution to the intermolecular interactions is represented by the so-called two-body energy term, which, in principle, could be suitably accounted for by dispersion-inclusive DFT methods. In particular, DFT-D*x* models were conceived as general-purpose corrections to Kohn–Sham DFT electronic energy for treating a large variety of chemical systems. Nevertheless, alternative strategies could be pursued for enhancing the accurate modeling of a given system of interest. In a recent work [12], we proposed a simple and system-specific computational protocol aimed at improving the description of non-covalent interactions by fine-tuning the DFT-D*x* models. The underlying idea of this approach was to purposely reoptimize the dispersion correction by tweaking the empirical parameters (i.e., scaling factors) of the Grimme’s D*x* multipole interaction potential towards the benchmark calculations, which were carried out on hundreds of non-equilibrium cluster configurations of growing size. The one-body energy contribution (i.e., monomer energy deviations) is factored out from the reference calculations to make the optimization of the dispersion correction more effective. As a result, the possible discrepancies between the DFT-D*x* models and reference data are suitably magnified and the system-size consistency could also be well scrutinized. The application of this approach has led to refined DFT-D*x* calculations that were in very good agreement with high-level quantum mechanical results, taking acetonitrile as a test case [12].

In this work, considering well-known DFT-D4 approximations, such as the generalized gradient approximation (GGA) (i.e., BLYP, PBE, and revPBE) and the B3LYP, PBE0, and revPBE0 hybrid functionals, we show how such models can be remarkably improved, when necessary, for accurately describing the intermolecular interactions between a large number of medium- to large-sized water clusters (i.e., 300 (H_2_O)*_n_* structures, *n* = 9, 15, and 27). As benchmark data, the quantum Monte Carlo (MC) calculations reported by Alfé et al. [13] were used for comparison. The results of the reoptimized B3LYP-D4, PBE-D4, and revPBE(0)-D4 models reported a notable (>4×) reduction in the mean absolute deviations (MAE per molecule) with respect to the default DFT-D4 throughout all the clusters considered, from 0.6–1.0 kcal/mol to about 0.1 kcal/mol. On the other hand, default BLYP-D4 was shown to already be rather satisfactory, while the uncorrected PBE0 was so impressively close to the reference data that the inclusion of dispersion correction led to inconsistent results. Moreover, a few water hexamer configurations, for which highly accurate data are available, were also considered for the sake of comparison. The results on the latter highlighted the limits of choosing small clusters for the careful assessment of DFT models aimed at predicting the properties of mesoscopic systems. Note that the main purpose of this work is not to present another “accurate” DFT model or provide a systematic comparative DFT study on water, since state-of-the-art DFT methodologies have already been reviewed elsewhere (see, for example, refs. [1,14]). Rather, this study supports the use of the present optimization procedure for attaining highly accurate DFT-D*x* models tailored towards specific molecular systems, in the case when standard all-round parametrization seems unsatisfactory.

## 2. Results

### 2.1. Assessment of the D4 Dispersion Energy Correction

**Figure 1:**
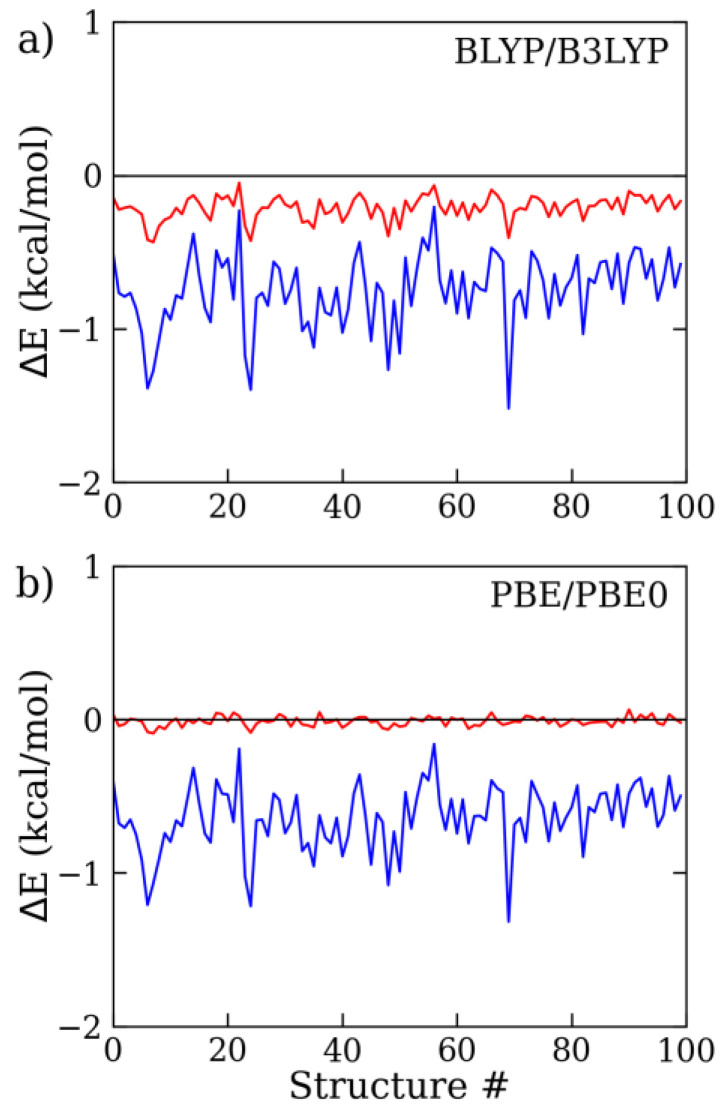
One-body energy deviation issuing from (**a**) BLYP (blue) and B3LYP (red), and from (**b**) PBE (blue) and PBE0 (red), using the aug-cc-pVTZ basis set with respect to the Partridge and Schwenke potential on a set of water monomer configurations (each structure identified by its own order number #) taken from the 9-mer water clusters (see Section 4 for further details).

A large set of water molecular clusters (i.e., about 300 configurations, (H_2_O)*_n_* with *n* = 6, 9, 15, and 27) was considered to assess the effect of Grimme’s D4 correction term on the intermolecular interaction energy computed at the BLYP, B3LYP, PBE, and PBE0 levels of theory, as compared to high-level MC calculations with a statistical error of ~5 meV (~0.1 kcal/mol) per monomer. The water cluster structures were taken from ref. [13] and were selected because they displayed a good representative ensemble of non-equilibrium configurations, which were characterized by variable sizes and compactness. For the sake of comparison, we also considered the water hexamer cluster, since it has been extensively investigated in past benchmarking studies [1,15]. To highlight the contribution of the one-body energy ($\Delta E_{1-body}^{}$,), in Figure 1, the DFT results for the water monomers taken from the 9-mer clusters are shown with respect to the reference data (for further details on the evaluation of the one-body energy term and the interaction energy term, see Methods section, Section 4). Note that, in this case, the reference data on the monomer deviation energies were evaluated from the accurate Partridge–Schwenke intramolecular potential (see Section 4). All the DFT approximations underestimated, to some degree, the water monomer energies, with the notable exception of PBE0, which showed an average monomer energy deviation of 0.02 kcal/mol (i.e., MAE). Both BLYP and PBE provided an average energy error of more than 0.5 kcal/mol (0.75 kcal/mol for BLYP and 0.64 kcal/mol for PBE), while the error was more contained for B3LYP (MAE: 0.21 kcal/mol). The observed discrepancies in the one-body energy could amount to about 30% or more of the overall total interaction energy (vide infra). For such a reason, we decided to focus our study on the analysis of the water intermolecular interactions beyond the one-body term, whose failure cannot be cured by dispersion-inclusive models. Hereafter, the energy deviations issuing from the water monomers ($\Delta E_{1-body}^{n}$) were systematically neglected from the computed interaction energy. The details of the present methodology are fully described in the Methods section (Section 4). Note that this has to be taken into account when comparing our results to similar DFT analyses presented elsewhere.

**Figure 2:**
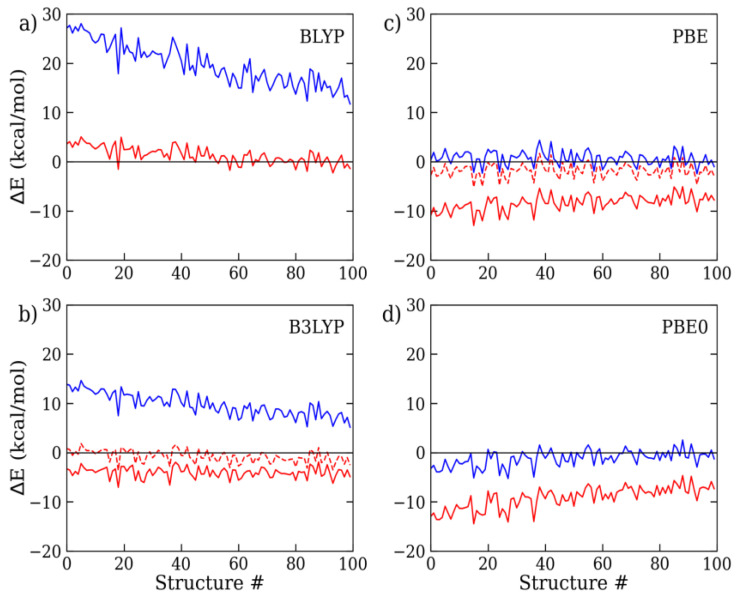
(**a**) BLYP, (**b**) B3LYP, (**c**) PBE, and (**d**) PBE0 interaction energy deviations with respect to MC, as computed with the aug-cc-pVTZ basis functions on a set of 9-mer water cluster configurations. Standard DFT results are depicted in blue, while default (solid line) and reoptimized (dashed line) DFT-D4 results are in red.

**Figure 3:**
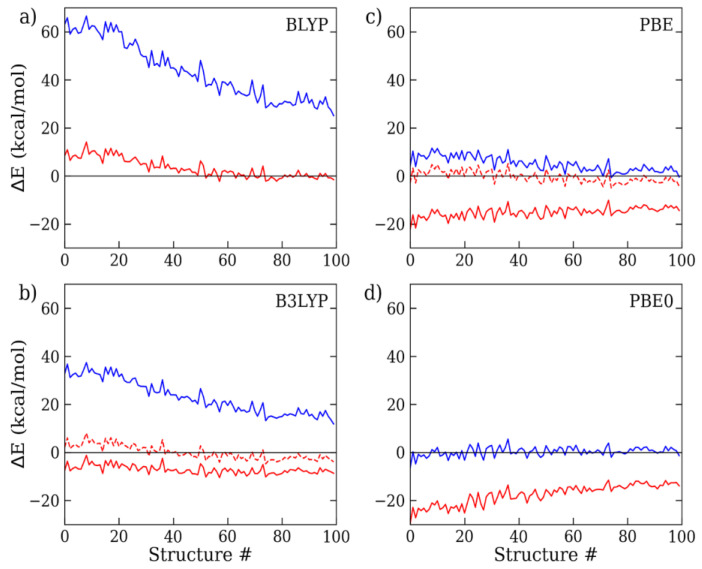
(**a**) BLYP, (**b**) B3LYP, (**c**) PBE, and (**d**) PBE0 interaction energy deviations with respect to MC, as computed with the aug-cc-pVTZ basis functions on a set of 15-mer water cluster configurations. Standard DFT results are depicted in blue, while default (solid line) and reoptimized (dashed line) DFT-D4 results are in red.

**Figure 4:**
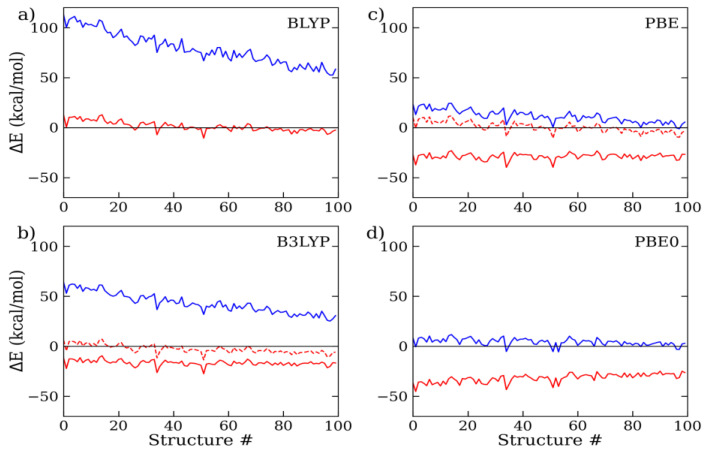
(**a**) BLYP, (**b**) B3LYP, (**c**) PBE, and (**d**) PBE0 interaction energy deviations with respect to MC, as computed with the aug-cc-pVTZ basis functions on a set of 27-mer water cluster configurations. Standard DFT results are depicted in blue, while default (solid line) and reoptimized (dashed line) DFT-D4 results are in red.

In all the DFT calculations, we consistently employed the extended aug-cc-pVTZ basis set throughout all the cluster sizes, since test calculations on the 9-mer cluster structures have shown that the residual error between the triple-ζ and quadruple-ζ basis functions is less than 0.02 kcal/mol per monomer (i.e., MAE/mol), or five times smaller than the intrinsic error of the reference MC data (~0.1 kcal/mol) (Figure S1). As a consequence, in the following, the basis set superposition error (BSSE), though present, was considered negligible with respect to the inaccuracy of the combined density functional/dispersion correction model. To assess the extent of the D4 correction, we evaluated the discrepancy between the standard (i.e., uncorrected DFT) and dispersion-corrected (i.e., DFT-D4) calculations with respect to the MC on a number of representative 9-mer, 15-mer, and 27-mer structures. The results are shown in Figure 2, Figure 3 and Figure 4 and Table 1. For all the considered exchange–correlation functionals, the addition of the semi-classical dispersion term led to an enhanced stabilization of the interaction energy, as expected, but the results for the different DFT approximations appeared to vary largely with respect to the MC reference data. Considering the 9 mer cluster (Figure 2), while the standard BLYP and B3LYP calculations underestimated the interaction energy to a great extent, by about 20 and 10 kcal/mol, respectively, the DFT-D4 results showed significant improvements. In particular, BLYP-D4 reported a remarkable reduction in the average energy deviation to less than 2 kcal/mol (MAE/mol: 0.19 kcal/mol), while the average discrepancy of B3LYP was reduced from 10 to 4 kcal/mol (MAE/mol: 0.45 kcal/mol). Then, upon the addition of the D4 correction, B3LYP reported a small overestimation of the water cluster binding energy. A rather similar trend was also observed for the 15-mer and 27-mer clusters (Figure 3 and Figure 4): overall, the BLYP-D4 results remained impressively close to the MC data, while B3LYP-D4 showed a small departure from the benchmark data (i.e., stronger interaction energy) going to the larger cluster (for the 27-mer cluster, MAE/mol: 0.62 kcal/mol). It is worth noting that the D4 contribution not only reduced the distance from the reference data, but also somewhat smoothed out the observed energy fluctuations (i.e., differences in energy deviations), making the overall profile more regular throughout all the configurations under scrutiny. This is relevant since the cluster structures were ordered according to their radius of gyration, with the former configurations being more compressed than the latter (as shown in Figure S2); therefore, they were prone to providing higher deviations, as previously seen in ref. [13]. For the sake of comparison, we also considered the older BLYP-D3 correction, since in several past studies on clusters and liquid water, this or even older variants of the Grimme’s approach were considered. We found that the D3 results were rather similar to those for D4, only slightly exceeding the cluster interaction energy: for the 9-mer cluster, the MAE/mol resulted 0.23 kcal/mol instead of 0.19 kcal/mol with the latest D4 variant, and we could then confirm a slight improvement in the latter.

On the contrary, the results issued from PBE and PBE0 on the same water clusters were qualitatively different from the previous DFT approximations. The uncorrected DFT calculations already showed a remarkable agreement with the MC data, especially for PBE0, while the corresponding dispersion-corrected results led to significantly overestimated interaction energies. The standard PBE0 showed an MAE per molecule of about 0.1 kcal/mol throughout all the clusters, while the addition of the D4 contribution brought the average energy deviations up by an order of magnitude (MAE/mol: ~1 kcal/mol). These findings seem in line with those hinted by the minimum-energy configurations of the water hexamer cluster, where both the Grimme’s DFT-D model and the Tkatchenko and Scheffler [16] method were tested, leading, overall, to an overstabilization of the cluster [15]. Since water interactions are mostly dominated by hydrogen bonding and polar electrostatic interactions, and to lesser extent by van der Waals interactions, the effect of the D4 correction could likely be overemphasized for the PBE(0) functional. Similar to PBE0, the energy deviations worsened upon the inclusion of the D4 semi-classical correction to PBE, reaching an MAE per molecule of ~1 kcal/mol, while the pristine DFT approximation showed an error of 0.1–0.3 kcal/mol per monomer. The fact that the uncorrected PBE and PBE0 results for the large water clusters were close to the benchmark and similar to one another seems to confirm the previous test calculations [11,13] on the selected structures from the WATER27 benchmark set [17], which includes a small number of optimized water cluster geometries: PBE provided better overall results than PBE-D3 and revPBE-D3 [11]. Additionally, the observed agreement between PBE and PBE0 could explain the reason why the ab initio molecular dynamics simulations of the liquid water reported undistinguishable radial distribution functions (RDFs) using such exchange–correlation functionals [18]. Despite these findings, which are in line with our results, both revPBE-D3 and revPBE0-D3 have recently been adopted for simulations of liquid water [10,19,20] and, hence, are considered superior to the corresponding parent functionals (i.e., PBE and PBE0). Here, for the sake of comparison, we also tested revPBE-D4 and revPBE0-D4, in addition to a recently developed effective DFT model derived from PBE0-D3, namely PBEh-3c [21]. The latter has the advantage of including three corrections all at once to account for the dispersion effects, short-range basis set superposition errors, and an effective small basis set. In contrast to PBE(0), the dispersion-inclusive revPBE(0)-D4 variants corrected the corresponding pristine exchange–correlation approximations well, but the revPBE-D4 binding energies remained underestimated (MAE/mol: ~0.7 kcal/mol), while revPBE0-D4 showed cluster energies that were more in line with the reference data (MAE/mol: 0.36 kcal/mol), as depicted in Figure S3 and reported in Table S1. On the other hand, the PBEh-3c results showed, overall, largely overbound cluster energies, accentuated in the case of the largest cluster (Figure S4).

### 2.2. DFT-D4 Optimization of Water Intermolecular Energy

The above analysis of the Grimme’s D4 dispersion correction on water clusters of growing sizes emphasized a variable effect when applied to some of the most popular GGA (i.e., BLYP, PBE, and revPBE) and hybrid (i.e., B3LYP, PBE0, and revPBE0) DFT approximations. The most satisfactory corrected approximation was found to be BLYP-D4, which showed, overall, a very good agreement with the reference data, while the B3LYP-D4 results were improved over the corresponding uncorrected DFT, yet not completely satisfactory. On the other hand, the standard PBE0 matched very well with the MC benchmark calculations, followed closely by PBE, without the need of any correction, which, in fact, caused a significant deterioration in the description of the water interaction energy. Both revPBE-D4 and revPBE0-D4 were also significantly improved over the corresponding standard DFT approximations. In light of these results, we set out to improve the performance of B3LYP-D4, PBE-D4, revPBE-D4, and revPBE0-D4 towards the reference data by fine tuning the D4 model (i.e., the *S_n_* scaling factors), according to the general approach presented in Section 4 that was successfully applied to acetonitrile in a recent study [12]. Since the *n* = 8 order term accounts for a good extent of the overall dispersion energy correction, we initially considered the re-optimization of the *S*_8_ scaling factor, which is one of the few empirically adjustable parameters of the Grimme’s D4 model. In Figure S5a, the MAE issuing from the B3LYP-D4 calculations on the 15-mer water cluster, with respect to the reference data, is depicted as a function of the *S*_8_ parameter. Note that, for B3LYP-D4, the default *S*_8_ value is 2.029. As shown in Figure S5a, a satisfactory MAE was obtained by reducing the scaling factor to about 1.0. Then, we tested whether such an optimized parameter could consistently improve the results for all the molecular samples considered in this work. The results are depicted in Figure 2b, Figure 3b and Figure 4b and show a remarkable match of the refined B3LYP-D4 data with respect to the MC (see also Table 2): the average energy deviations (i.e., MAE/mol) decreased by a factor of 5 from ~0.5 kcal/mol to 0.1 kcal/mol. Turning to PBE-D4, since the discrepancy with respect to the MC was substantial, we could not solely adjust the *S*_8_ parameter in a suitable way. Indeed, the binding energies from the uncorrected PBE were already good enough that the missing dispersion was expected to be rather small. In this case, after some testing, we explored the possibility of greatly reducing the D4 correction by decreasing the contribution of the *n* = 6 order term and removing the *n* = 8 order one altogether. The obtained results showed again a quite satisfactory description of the water interaction energy throughout all the considered clusters, with an MAE/mol of ~0.1 kcal/mol (see Figure 2c, Figure 3c and Figure 4c and Table 2). The latter results were obtained by setting *S*_6_ = 0.4 and *S*_8_ = 0.0, where the default values for PBE were *S*_6_ = 1.0 and *S*_8_ = 0.959 (Figure S5b). Note that we are well aware that the *n* = 6 order term was theoretically derived to ensure the asymptotically correct behavior of the dispersion interaction and the general rule suggests to keep its contribution unmodified (i.e., *S*_6_ = 1.0). The present correction was introduced just to illustrate the kind of improvements an ad hoc refinement could achieve. To proceed further, we carried out a similar refinement of both revPBE-D4 and revPBE0-D4 by optimizing the corresponding *S*_8_ parameter, which had to be increased, as shown in Figure S5c,d (for revPBE-D4, from *S*_8_ = 1.746 to 2.346 and for revPBE0-D4, from *S*_8_ = 1.571 to 2.071). Again, in both cases, we obtained an excellent level of agreement throughout all the clusters under consideration, as shown in Figure S3 and Table 2 (MAE/mol of ~0.1 kcal/mol).

### 2.3. Analysis of the Interaction Energy of Water Hexamer Clusters

**Figure 5:**
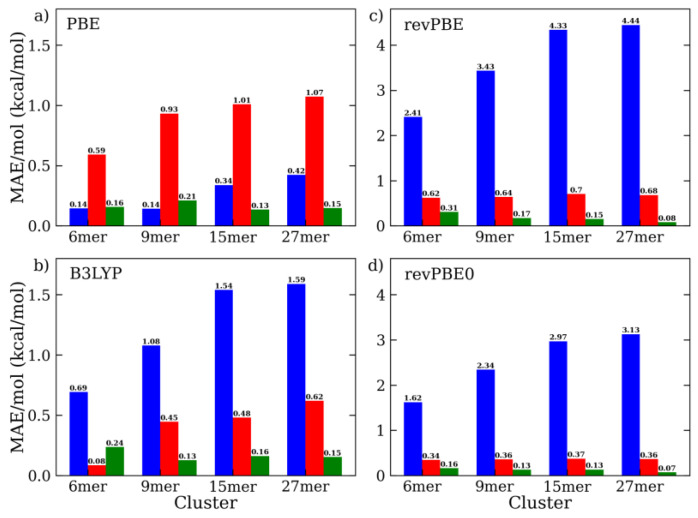
Mean absolute error (MAE) per molecule of (**a**) PBE, (**b**) B3LYP, (**c**) revPBE, and (**d**) revPBE0 using aug-cc-PVTZ basis set interaction energy with respect to MC, as computed by standard DFT (blue) and DFT-D4 (default in red, optimized in green) on water clusters of growing size (i.e., from 6-mer to 27-mer).

At this point, it is interesting to note that calculations on small molecular clusters, such as dimers and hexamers, are sometimes used to make predictions about the quality of liquid bulk properties, though this could lead to a misleading interpretation. As an illustrative example of this issue, we consider here the case of the water hexamer cluster. This is a particularly well-studied system in the literature, since it represents the first water assembly showing a number of different three-dimensional minimum-energy configurations, among which there is subtle competition between the extended structures (i.e., the book and ring configurations), displaying fewer but stronger hydrogen bonds and compact structures (i.e., the prism and cage configurations) that display more but weaker hydrogen bonds (see Figure S6). In analogy with our previous analysis, we compared the water binding energies (i.e., without the one-body contribution) issuing from the same DFT and DFT-D4 models investigated above on four hexamer configurations (Figure S7). With some exceptions, the results on such small clusters did not account well for the observations made for more extended molecular systems (Figure 5 and Figure S8). For instance, BLYP and B3LYP underestimated the errors observed in the larger systems: for BLYP, the MAE/mol was 1.43 kcal/mol for 6-mer, but 2.9 kcal/mol for 15-mer and 27-mer, and for B3LYP, the MAE/mol was 0.69 kcal/mol for 6-mer, but 1.6 kcal/mol for 27-mer. Similarly, the PBE-D4 and PBE0-D4 results for the 6-mer cluster provided average errors (MAE/mol ~0.6 kcal/mol) that were well below those obtained for the more extended systems (~1 kcal/mol). On the other hand, BLYP-D4 and PBE severely overestimated the same deviations: for BLYP-D4, the MAE/mol was 0.62 kcal/mol for 6-mer and 0.14 kcal/mol for 27-mer, and for PBE, the MAE/mol was 0.14 kcal/mol for 6-mer and 0.42 kcal/mol for 27-mer. Remarkably, we observed that the reoptimized B3LYP-D4, PBE-D4, revPBE-D4, and revPBE0-D4 models seemed to provide consistent results throughout all the clusters under study, from 6-mer to 27-mer, though this could be partially fortuitous (Figure 5). Additionally, it is worth noting that the refined revPBE-D4 and revPBE0-D4 models reported the correct order of stability of the water hexamer configurations (Prism < Cage ≈ Book < Ring), in addition to a satisfactory agreement with the MC data (Figure S7).

## 3. Discussion

In this study, we showed how a recently proposed system-dependent optimization protocol could be applied to develop improved dispersion-corrected DFT models based upon the latest Grimme’s DFT-D4 semi-classical scheme for the accurate description of water interaction energy. The main idea of this approach was to refine the dispersion correction towards accurate QM calculations for multiple non-equilibrium cluster structures of growing size. In our view, the present strategy allows us to better assess the performances of specific DFT approximations by highlighting the relative advantages and pitfalls for treating extended mesoscopic systems. In fact, the increase in the mean absolute errors with respect to the benchmark MC data, evaluated as a function of the cluster size, was rather apparent in all cases. It is worth noting that, in the present analysis, the water intermolecular interactions were obtained by removing the so-called one-body energy, i.e., the contribution to the energy deviations issuing from geometry distortions of the individual water molecules. This was motivated by the observation that the latter is often non-negligible with respect to the total intermolecular energy, especially for GGA functionals, thus preventing a proper assessment of the dispersion interaction energy, and, more generally, any beyond the one-body interaction. First, taking into consideration some popular exchange–correlation models, namely BLYP, B3LYP, PBE, PBE0, revPBE, and revPBE0, our results have helped to highlight the effect of a lack of dispersion interaction and its recovery through an inclusion of the D4 correction. Specifically, the D4 contribution, in its standard parametrization, was shown to very satisfactorily cure the performance of BLYP (i.e., BLYP-D4) throughout all the water clusters considered in this study, in line with previous studies on water clusters and liquid water that have indicated the beneficial effect of dispersion inclusion. On the other hand, the B3LYP-D4 model provided results that were much less satisfactory, and both PBE-D4 and PBE0-D4 showed some clear issues, with no obvious relation with the type of approximation, whether it was GGA or hybrid. The uncorrected PBE0 matched very well with the reference data, suggesting that the presumed lack of dispersion, as previously reported, could have been somehow overemphasized for the case of water, likely due to the primary role played by the hydrogen bonding over the van der Waals interactions. PBE was also rather close to the MC results, which may explain the surprising similarities observed on the RDFs of the liquid water issuing from the PBE and PBE0 ab initio MD simulations [18]. Nevertheless, upon the re-optimization of a few parameters, we noted a remarkable improvement in both B3LYP-D4 and PBE-D4 with respect to the reference data at any cluster size, reaching an MAE per molecule of ~0.1 kcal/mol, with the energy deviations being effectively minimized by a factor of four or higher from the standard D4 parametrization. Similarly, we showed how further refinements of the already good revPBE-D4 and revPBE0-D4 models led to remarkable results for the computed interaction energy of the water clusters in comparison to the MC. We believe these findings prove once more the general applicability of the present simple approach for the system-specific optimization of DFT-D4 models, which can be easily extended to other popular functionals.

## 4. Methods

The optimization procedure for the dispersion correction term closely followed the one originally described in ref. [12]. Briefly, according to Grimme’s model, the standard Kohn–Sham DFT electronic energy ($E_{DFT}$) was corrected by the addition of a dispersion energy term treated as a semi-classical (i.e., independent of the electronic structure) interatomic potential, including two or more high-order multipole interaction terms (typically, C_6_/R^6^, C_8_/R^8^, and so on), which were modulated by further damping functions and scaling factors. The total energy is described as (neglecting the three-body or higher-order terms):

(1) $$E_{DFT-D}=E_{DFT}-E_{dis}$$

where *E_dis_* is expressed as:

(2) $$E_{dis}=\overset{N}{\underset{a,b}{\sum }}\overset{}{\underset{n=6,8,\ldots }{\sum }}S_{n}\frac{C_{n}^{ab}}{r_{ab}^{n}}f_{d,n}\left(r_{ab}\right)$$

where $C_{n}^{ab}$ is the *n*-th order dispersion coefficient (orders *n* = 6, 8, …) defined for any given atom pair (*a*, *b*) in the system, $r_{ab}$ is the internuclear atom pair distance, $f_{d,n}\left(r_{ab}\right)$ is a damping function introduced to avoid singularities at small interatomic distances, and $S_{n}$ are scaling factors (typically dependent on the DFT method). For a detailed discussion of the meaning and definition of all the parameters, see ref. [5]. In practical implementations, the *n*-th order is usually truncated after *n* = 8 and most of the parameters are computed ab initio $\left(C_{6}^{ab}\right)$, derived recursively $\left(C_{8}^{ab}\right)$, or kept fixed (e.g., *S*_R,8_ and *S*_6_ are set to 1 for all the DFT methods, except those accounting for the dispersion energy). On the other hand, the *S*_8_ scaling factor is regarded as an empirical parameter, among others, and is adjusted to implicitly account for higher multipolar terms beyond the dipole–dipole contribution. As such, the *S*_8_ scaling factor is generally dependent upon the specific DFT approximations. As proposed in ref. [12], the *S*_8_ parameter can be conveniently refined against available benchmark data on molecular clusters of growing size as an effective computational strategy aimed at better assessing the performance of DFT-D models. Accordingly, in the present study, we refined this scaling factor so as to minimize the energy deviations in the interaction energies between the MC and DFTs, as issued from calculations on a large set of water clusters. For each cluster configuration, such an interaction energy deviation is defined by subtracting the one-body energy deviation, $\Delta E_{1-body}^{n}$, from the total interaction energy difference, $\;\Delta E^{n}$, as follows:

(3) $$\Delta E^{n}-\Delta E_{1-body}^{n}$$

where:

(4) $$\Delta E^{n}=\left(\Delta E_{DFT}^{n}-\Delta E_{DMC}^{n}\right)\;\Delta E_{1-body}^{n}=\left(\Delta E_{1-body,DFT}^{n}-\Delta E_{1-body,DMC}^{n}\right)$$

while for the one-body and total interaction energy of the corresponding electronic structure calculation, we have:

(5) $$\Delta E_{X}^{n}=E_{X}^{n}-nE_{X}^{ref}\;\Delta E_{1-body,X}^{n}=\overset{n}{\underset{i}{\sum }}\left(E_{X}^{i}-E_{X}^{ref}\right)$$

where $E_{X}^{n}$ is the total energy of the *n*-th cluster (with *n* = 6–27) configuration computed at the *X* (=MC, DFT, DFT-D4) level of theory, $E_{X}^{ref}$ is the energy of the isolated molecule at the reference gas-phase geometry (i.e., OH = 0.95865 Å and HOH = 104.348°) computed at the same level of theory, and $E_{X}^{i}$ is the energy of the isolated *i*-th (with *i* = 1-*n*) molecule (possibly distorted) taken from the cluster configuration.

The water cluster configurations of growing size (i.e., 300 (H_2_O)*_n_* structures, *n* = 9, 15, and 27) and the corresponding reference MC calculations of the total interaction energies were obtained from ref. [13]. Furthermore, the reference calculations and structures on a few optimized geometries of the hexamer clusters were taken from ref. [15]. In order to obtain the intermolecular interaction energy from the total interaction energy (Equations (3)–(5)), we computed the one-body energy term ($\Delta E_{1-body}^{n}$) from the accurate single-molecule potential energy derived by Partridge and Schwenke [22], using an in-house code. The single-point energy calculations at the DFT level of theory on the water molecular clusters were carried out with the Gaussian16 [23] software package, using a combination of Becke’s exchange functional [24] with the correlation function LYP [25,26] (i.e., BLYP), the Perdew–Burke–Ernzerhof (PBE) [27] functional, and the B3LYP [28,29] and PBE0 [30,31] hybrid functionals. The test calculations were carried out with the Dunning’s correlation basis sets, aug-cc-pVDZ, aug-cc-pVTZ, and aug-cc-pVQZ [32]. The dispersion correction energies were evaluated using Grimme’s D4 [6,7] model, as implemented in the DFT-D4 standalone code available on GitHub. In addition, the PBEh-3c [21] composite scheme, which uses a modified hybrid parameterization of the PBE exchange–correlation functional combined with the geometrical counter-poise correction (gCP) [33] and the D3 dispersion correction [5] using the Becke–Johnson dumping [34] as implemented in Orca 5.0.4 [35], was tested for comparison with the other DFT approximations. Similarly, the revPBE [36] and revPBE0 functionals with the D4 correction [5,34] were also tested. The data analysis and plotting were performed using in-house python codes and the molecular structures representation was obtained using the VMD software package [37].

## 5. Conclusions

As already noted in a previous study on acetonitrile [12], the present findings promptly suggest that the dispersion-inclusive DFT models reporting the best results on large water clusters may also show an improved description of the liquid phase (e.g., equilibrium density, radial distribution functions, and chemical potentials, etc.). Yet, it should be noted that first-principle simulations of liquids could be also affected by various theoretical and technical issues absent in cluster studies, such as the problems of finite system size, equilibration convergence, and so on, in addition to the proper treatment of the dispersion interactions. In addition, recent advances have better assessed the non-negligible and critical role of nuclear quantum effects in molecular liquids such as water [10,20], which in fact could be more relevant than previously predicted [38]. In this regard, we think that a generally safe approach would recommend preliminary tests of the electronic structure method of choice on large sets of medium to large molecular clusters before an application to condensed phase systems. On a further note, it is interesting to consider the versatility of the present optimization protocol for pursuing ad hoc and distinct refinements of Grimme’s dispersion corrections, in order to apply it to solute-solvent systems and, more generally, solutions and mixtures in which only selective intermolecular interactions are corrected, whenever necessary.

## Figures and Tables

**Table 1:** Error (Err), mean square error (MSE), mean absolute error (MAE), and MAE per molecule (MAE/molecule) of B3LYP, BLYP, PBE, and PBE0, using the aug-cc-PVTZ basis set interaction energy deviations with respect to MC, as computed by using standard DFT and default DFT-D4 on water clusters of growing size (i.e., from 9-mer to 27-mer) considered in this study.

Cluster		Err/ kcal mol^−1^	MSE/ kcal mol^−1^	MAE/ kcal mol^−1^	MAE per Molecule/ kcal mol^−1^
**9-mer**					
	BLYP	19.7	405.77	19.7	2.19
	BLYP-D4	1.33	4.65	1.72	0.19
	B3LYP	9.71	99.4	9.71	1.08
	B3LYP-D4	−4.02	17.21	4.02	0.45
	PBE	0.7	2.42	1.27	0.14
	PBE-D4	−8.39	72.84	8.39	0.93
	PBE0	−1.21	4.07	1.56	0.17
	PBE0-D4	−9.2	89.66	9.2	1.02
**15-mer**					
	BLYP	43.58	2044.31	43.58	2.91
	BLYP-D4	3.329	26.95	3.82	0.25
	B3LYP	23.08	581.8	23.08	1.54
	B3LYP-D4	−7.19	54.89	7.19	0.48
	PBE	5.05	34.93	5.08	0.34
	PBE-D4	−15.13	233.15	15.13	1.01
	PBE0	0.28	3.61	1.45	0.1
	PBE0-D4	−17.59	325.94	17.59	1.17
**27-mer**					
	BLYP	79.1	6503.64	79.1	2.93
	BLYP-D4	1.24	23.58	3.69	0.14
	B3LYP	42.92	1936.93	42.92	1.59
	B3LYP-D4	−16.7	286.26	16.7	0.62
	PBE	11.38	165.4	11.41	0.42
	PBE-D4	−28.93	845.73	28.93	1.07
	PBE0	4.29	30.44	4.76	0.18
	PBE0-D4	−31.9	1035.2	−31.9	1.18

**Table 2:** Error (Err), mean square error (MSE), mean absolute error (MAE), and MAE per molecule (MAE/molecule) of B3LYP, PBE, revPBE, and revPBE0 using the aug-cc-PVTZ basis set interaction energy deviations with respect to MC, as computed by using optimized DFT-D4 on water clusters of growing size (i.e., from 9-mer to 27-mer) considered in this study.

Cluster		Err/ kcal mol^−1^	MSE/ kcal mol^−1^	MAE/ kcal mol^−1^	MAE per Molecule/ kcal mol^−1^
**9-mer**					
	PBE-D4	−1.8	5.06	1.9	0.21
	B3LYP-D4	−0.75	2.03	1.14	0.13
	revPBE-D4	1.31	3.32	1.55	0.17
	revPBE0-D4	0.60	1.99	1.16	0.13
**15-mer**					
	PBE-D4	−0.40	5.69	2.02	0.13
	B3LYP-D4	−0.03	8.35	2.42	0.16
	revPBE-D4	0.92	7.2	2.26	0.15
	revPBE0-D4	−0.09	6.16	1.97	0.13
**27-mer**					
	PBE-D4	0.57	24.12	3.97	0.15
	B3LYP-D4	−2.8	23.92	4.12	0.15
	revPBE-D4	0.75	8.31	2.14	0.08
	revPBE0-D4	−0.83	7.89	1.9	0.07

## Data Availability

All data available upon request.

## Supplementary Materials

The following supporting information can be downloaded at: https://www.mdpi.com/article/10.3390/molecules28093834/s1, Figure S1: Basis set comparison on a subset of 9 mer water cluster configurations; Figure S2: Squared radius of gyration of three sets of water clusters of growing size; Figure S3: Interaction energy deviations with respect to MC for the revPBE and revPBE0 on a set of 9 mer, 15 mer and 27 mer water cluster configurations; Table S1: Error, mean square error, mean absolute error, and MAE per molecule of revPBE and revPBE0 on a set of 9 mer, 15 mer and 27 mer water cluster configurations; Figure S4: Interaction energy deviations with respect to MC for the PBEh-3c composite scheme on a set of water clusters of growing size; Figure S5: DFT-D4 optimization procedure for PBE, B3LYP, revPBE and revPBE0; Figure S6. Representation of prism, cage, book and ring 6 mer molecular structures; Figure S7: Interaction energy/molecule on a set of 6 mer water cluster configurations; Figure S8: MAE/molecule with respect to MC on water clusters of growing size (i.e., from 6 mer to 27 mer).
