# Supplementary material for: Fine Tuning the Intermolecular Interactions of Water Clusters Using the Dispersion-Corrected Density Functional Theory
Source: Molecules. 2023 Apr 30;28(9):3834. doi: 10.3390/molecules28093834 (PMC10180381; doi:10.3390/molecules28093834)
Supplement: Supplementary file 1 [file molecules-28-03834-s001.zip › molecules-2356823-supplementary.pdf]

Supplementary Materials for

Fine Tuning the Intermolecular Interactions of Water Clusters using Dispersion-Corrected Density Functional Theory

Alfonso Ferretti,[1,2] Laura Canal,[3] Robert A. Sorodoc,[3] Sourab Sinha[1] and Giuseppe Brancato[1,2]*

1 Scuola Normale Superiore and CSGI, Classe di Scienze, Piazza dei Cavalieri 7, I-56126 Pisa, Italy

2 Istituto Nazionale di Fisica Nucleare (INFN) Sezione di Pisa, Largo Bruno Pontecorvo 3, I-56127 Pisa, Italy

3 Dipartimento di Ingegneria Civile ed Industriale, Università di Pisa, Largo Lucio Lazzarino 2, I-56124 Pisa, Italy

* Correspondence: giuseppe.brancato@sns.it

Content

1) **Figure S1.** Basis set comparison on a subset of 9mer water cluster configurations.
2) **Figure S2.** Squared radius of gyration of three sets of water clusters of growing size.
3) **Figure S3.** Interaction energy deviations with respect to MC for the revPBE and revPBE0 on a set of 9mer, 15mer and 27mer water cluster configurations.
4) **Table S1.** Error, mean square error, mean absolute error, and MAE per molecule of revPBE and revPBE0 on a set of 9mer, 15mer and 27mer water cluster configurations.
5) **Figure S4.** Interaction energy deviations with respect to MC for the PBEh-3c composite scheme on a set of water clusters of growing size.
6) **Figure S5.** DFT-D4 optimization procedure for PBE, B3LYP, revPBE and revPBE0.
7) **Figure S6.** Representation of prism, cage, book and ring 6mer molecular structures.
8) **Figure S7.** Interaction energy/molecule on a set of 6mer water cluster configurations.
9) **Figure S8.** MAE/molecule of BLYP and PBE0 with respect to MC on water clusters of growing size (i. e. from 6mer to 27mer).

Figure S1:

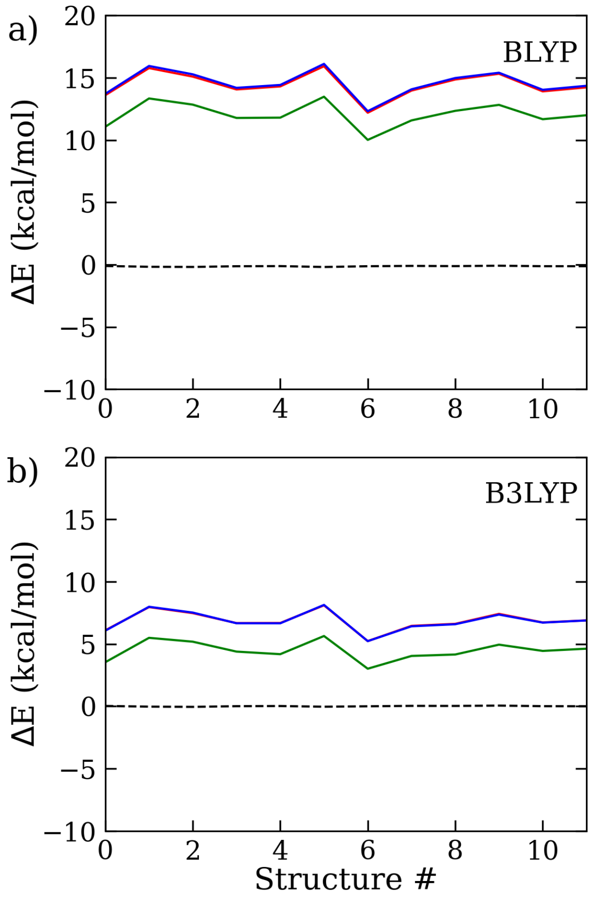

Figure S1 a) BLYP and b) B3LYP interaction energy deviations with respect to MC as computed with the aug-cc-pVDZ (green), aug-cc-pVTZ (blue) and aug-cc-pVQZ (red) on a subset of 9mer water cluster configurations (note that the structures reported here are not ordered according to ascending radius of gyration). The energy differences $\Delta\Delta E$(aug-cc-PVQZ - aug-cc-PVTZ) (dashed black) are also reported.

Figure S2

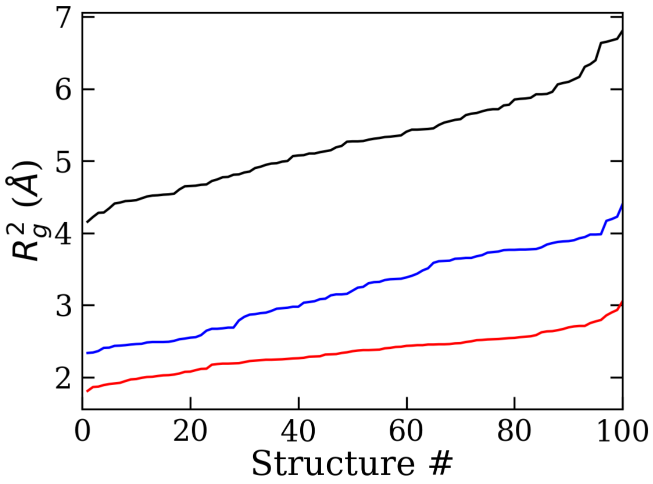

Figure S2 Squared gyration radius of water clusters of growing size 9mer (red), 15mer (blue) and 27mer (black).

Figure S3:

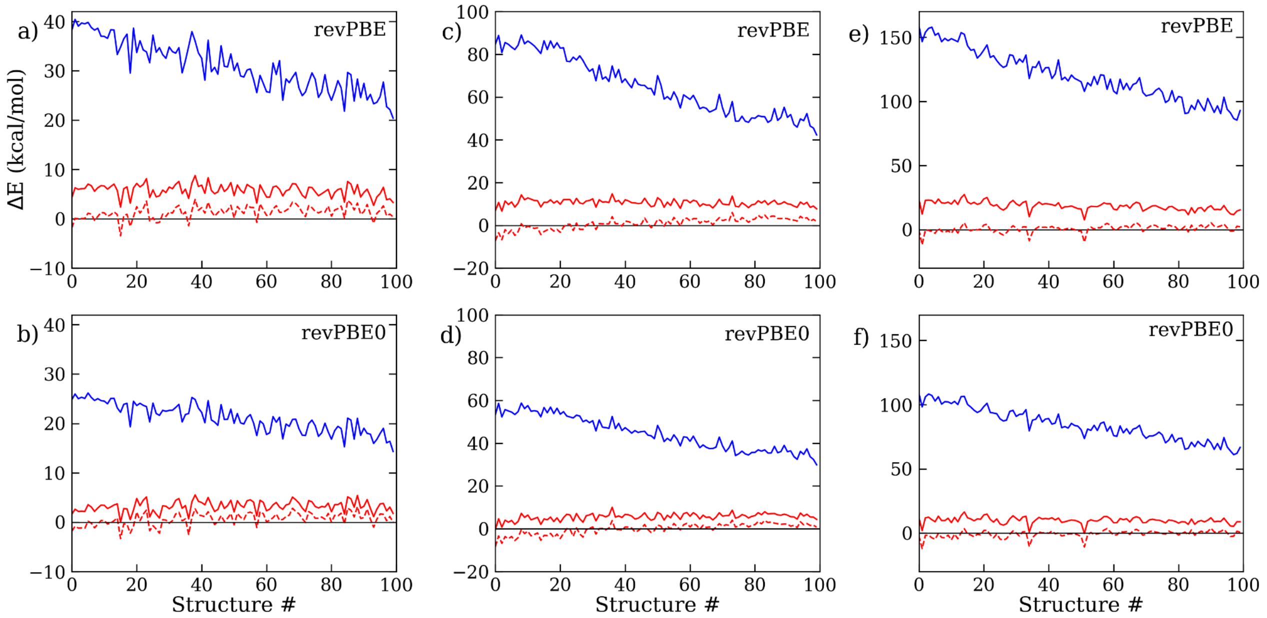

Figure S3 revPBE and revPBE0 interaction energy deviations with respect to MC as computed with the aug-cc-pVTZ basis functions on a set of 9mer (panels a) and b)), 15mer (panels c) and d)), 27mer (panels e) and f)) water cluster configurations. Standard DFT results are depicted in blue, while default (solid line) and optimized (dashed line) DFT-D4 results are in red.

Table S1:

Table 1. Error (Err), mean square error (MSE), mean absolute error (MAE), and MAE per molecule (MAE/molecule) of revPBE and revPBE0 using the aug-cc-PVTZ basis set interaction energy deviations with respect to MC as computed by using standard DFT and by using default DFT-D4 on water cluster of growing size (i.e., from 9mer to 27mer) considered in this study.

Cluster		Err/ kcal mol^{-1}	MSE/ kcal mol^{-1}	MAE/ kcal mol^{-1}	MAE per molecule/ kcal mol^{-1}
**9mer**					
	revPBE	30.91	980.3	30.91	3.43
	revPBE-D4	5.76	34.69	5.76	0.64
	revPBE0	21.06	451.38	21.06	2.34
	revPBE0-D4	3.20	11.54	3.20	0.36
**15mer**					
	revPBE	65.02	4407	65.02	4.33
	revPBE-D4	10.55	113.5	10.55	0.7
	revPBE0	44.54	2044.25	44.54	2.97
	revPBE0-D4	5.57	33.61	5.58	0.37
**27mer**					
	revPBE	120	14768	120	4.44
	revPBE-D4	18.37	348.66	18.37	0.68
	revPBE0	84.40	7276.38	84.40	3.12
	revPBE0-D4	9.81	102.7	9.82	0.36

Figure S4:

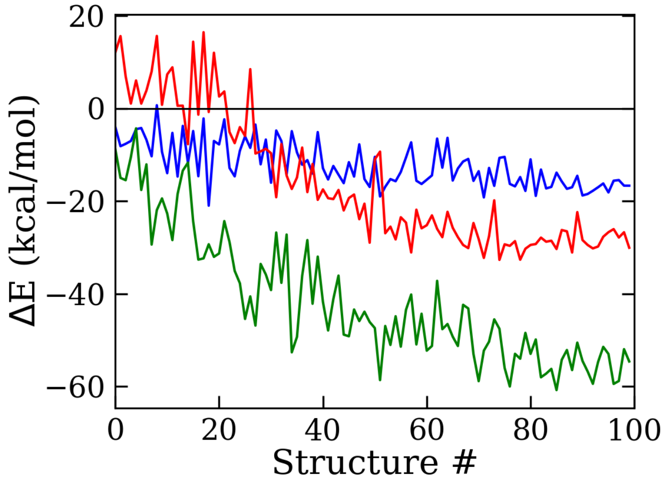

Figure S4 PBEh-3c composite scheme (see J. Chem. Phys. 143, 054107 (2015) for details on the functional and basis set) interaction energy deviations with respect to MC on a set of water cluster configurations of growing size. Results for the 9mer water cluster are depicted in blue, 15mer results are in red, and 27mer results in green.

Figure S5:

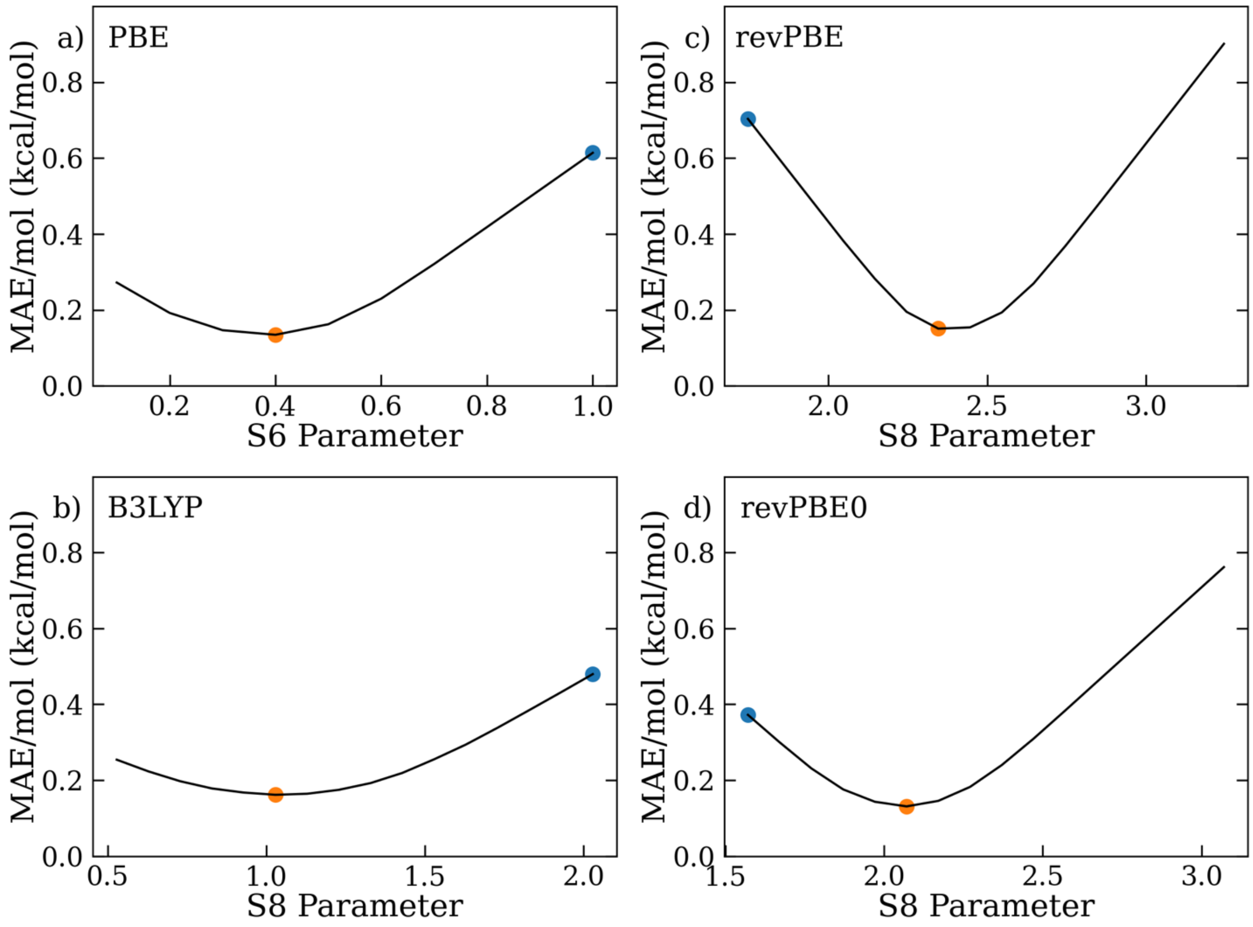

Figure S5 Mean absolute error (MAE) per molecule of a) PBE, b) B3LYP, c) revPBE and d) revPBE0 using the aug-cc-PVTZ basis set interaction energy with respect to MC as computed by DFT-D4 as a function of the S8 parameter for B3LYP, revPBE and revPBE0 and of the S6 Parameter for PBE (setting S8=0) on 15mer water cluster configurations. Default S8 and S6 parameters are highlighted as a blue dot, while optimized parameters are highlighted as an orange dot.

Figure S6:

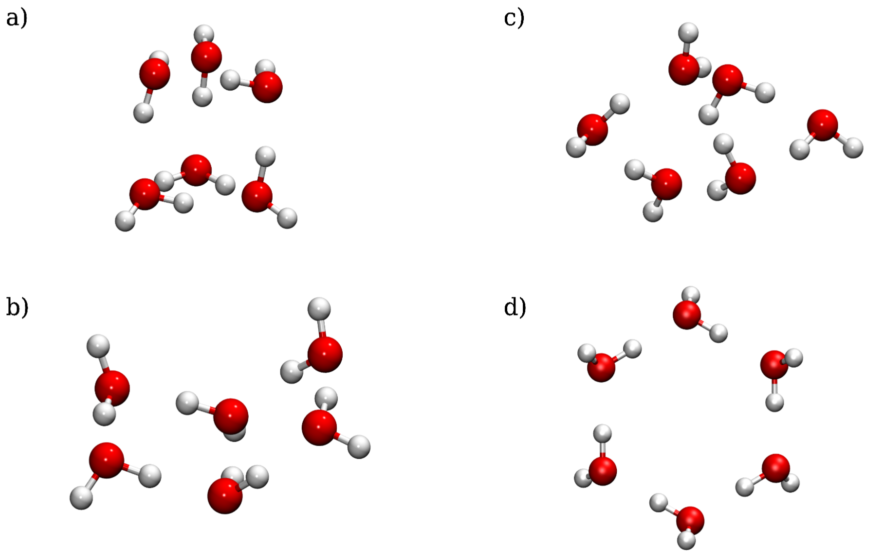

Figure S6: a) Prism, b) book, c) cage and d) ring water hexamer structures as obtained from J. Chem. Phys. 129, 194111 (2008). The structures were optimized at the MP2 level of theory using the aug-cc-pVTZ basis set.

Figure S7:

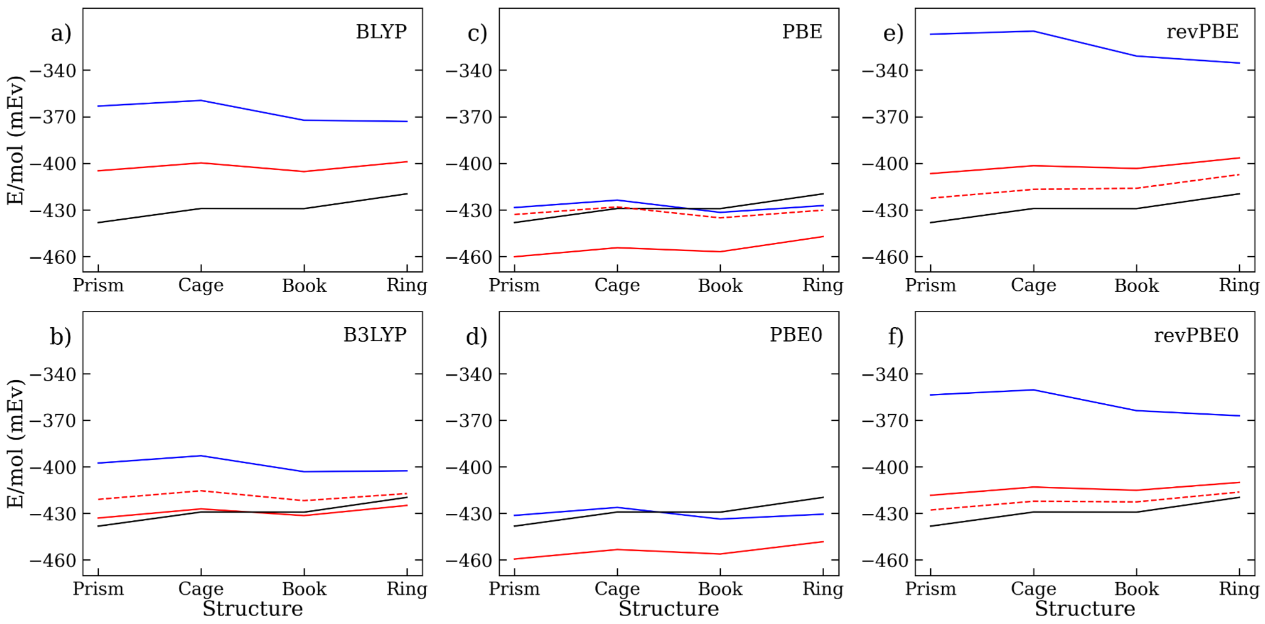

Figure S7 a) BLYP, b) B3LYP, c) PBE, d) PBE0, e) revPBE and f) revPBE0 interaction energy per molecule as computed with the aug-cc-pVTZ basis functions on four selected water hexamer configurations (see Figure S6). Reference MC results are in black, standard DFT results are depicted in blue, default (solid line) and reoptimized (dotted line) DFT-D4 results are in red.

Figure S8:

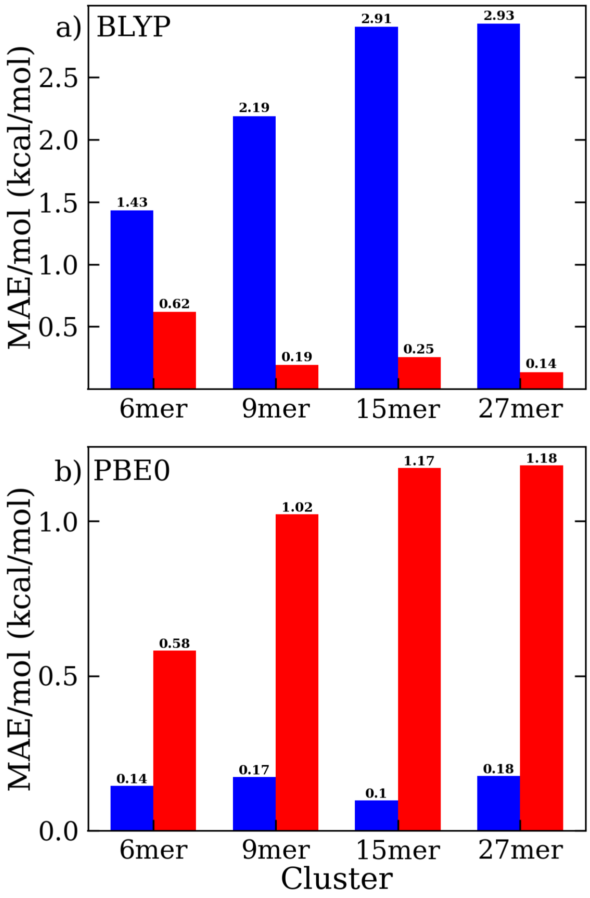

Figure S8: Mean absolute error (MAE) per molecule of a) BLYP and b) PBE0 using aug-cc-PVTZ basis set interaction energy with respect to MC as computed by Standard DFT (blue) and by DFT-D4 (default in red) on water clusters of growing size (i.e., from 6mer to 27mer).
